# Mixture models for analysis of melting temperature data

**DOI:** 10.1186/1471-2105-9-370

**Published:** 2008-09-11

**Authors:** Christoffer Nellåker, Fredrik Uhrzander, Joanna Tyrcha, Håkan Karlsson

**Affiliations:** 1 Department of Neuroscience, Karolinska Institutet, Retzius Väg 8 B2:5, 17177 Stockholm, Sweden; 2 Mathematical Statistics, Stockholm University, Kräftriket Hus 6, 106 91 Stockholm, Sweden

## Abstract

**Background:**

In addition to their use in detecting undesired real-time PCR products, melting temperatures are useful for detecting variations in the desired target sequences. Methodological improvements in recent years allow the generation of high-resolution melting-temperature (T_m_) data. However, there is currently no convention on how to statistically analyze such high-resolution T_m _data.

**Results:**

Mixture model analysis was applied to T_m _data. Models were selected based on Akaike's information criterion. Mixture model analysis correctly identified categories in T_m _data obtained for known plasmid targets. Using simulated data, we investigated the number of observations required for model construction. The precision of the reported mixing proportions from data fitted to a preconstructed model was also evaluated.

**Conclusion:**

Mixture model analysis of T_m _data allows the minimum number of different sequences in a set of amplicons and their relative frequencies to be determined. This approach allows T_m _data to be analyzed, classified, and compared in an unbiased manner.

## Background

Real-time PCR or semiquantitative PCR is widely used to detect and quantify specific target sequences. The exponential amplification of a sequence is monitored in real time by fluorescence. Commonly, a nonspecific fluorescent dye is used, such as SYBR Green I or LCGreen, which only reports the presence of double-stranded DNA. These dyes do not distinguish sequences and can thus report the amplification of undesired targets. Undesired sequences are normally detected during a dissociation step after thermocycling is complete. During dissociation, the double-stranded PCR products melt into single strands, so fluorescence is diminished. A curve can be produced by plotting the loss of fluorescence against a gradual increase in temperature. The temperature at which the rate of signal loss is the greatest can be defined as the melting temperature (T_m_) of the PCR product. Although the T_m _is sequence dependent, different sequences do not necessarily have different T_m_. However, the converse is true. The detection of different T_m _does imply the presence of different sequences. Therefore, by monitoring T_m_, we can distinguish different targets for one set of primers. This technique has been used for the detection of single-nucleotide polymorphisms [1], allelic discrimination [2], and strain typing of microorganisms [3-5]. We previously reported the use of T_m _analysis to detect the expression patterns of transcripts containing different members of the W family of human endogenous retrovirus (HERV) elements *in vitro *and *in vivo *[6,7].

The precision of the T_m _measurements determines the sensitivity with which different sequences can be distinguished. The instrument used to obtain the T_m _recordings is the principal factor limiting the amount of information that can be extracted from the data. We recently reported a method that allows improved resolution, reduced spatial bias, and automated data collection for T_m _detection in an ABI Prism 7000 Sequence Detection System (Applied Biosystems, Palo Alto, CA) [8]. Using a temperature indicator probe (T_m_probe) and an algorithm (GcTm) to interpolate more-precise T_m _measurements from multiple data points, the standard deviation of the measurement error (*σ*) of the T_m _recordings was improved from 0.19°C to 0.06°C [8].

However, there is no convention on how to analyze T_m _data to objectively distinguish sequences by T_m_. The need for such a tool becomes apparent when the T_m _data are: i) not easily stratified because of overlapping clusters of T_m _observations, and/or ii) if the number of different sequences and possible T_m _categories are unknown. In this report, we use mixture model analysis to construct a model for a particular set of primer targets, to classify T_m _data, and to calculate the mixing proportions of the amplicons within these categories. The mixture model technique allows T_m _analysis to be applied to any set of primers to determine the minimum number of T_m _categories (i.e., the number of different sequences detected) and the mixing proportions (frequency distributions) of the detected categories. Thus, mixture model analysis of T_m _data is an objective method with which more refined T_m _assays can be established.

## Results

**Figure 1:**
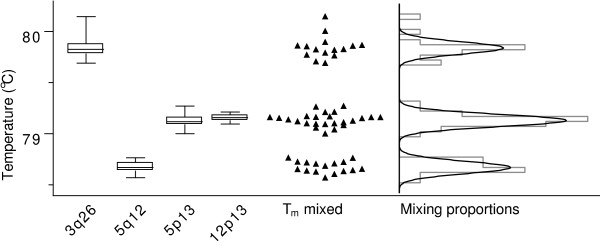
**T_m _profiles for four individual plasmid targets with known sequences and their analysis in a mixed population by mixture model analysis.** Box and whisker plots represent the T_m _(median and range) of the four known sequences amplified separately. Dot plot shows the mixed population of the four T_m _groups. Horizontal bar graph and Gaussian curve plot represent the grouped raw data of the T_m _and the categories, respectively, with mixing proportions determined by mixture model analysis. The mixture models determined the mixing proportions of the three categories to be 15, 24, and 16, which exactly matched the proportions of the different sequences used.

In a T_m _analysis using the T_m_probe and GcTm program, described previously [8], we demonstrated, using plasmids containing known sequences, that it was possible to distinguish some but not all sequences based on their T_m_. In the present report, we applied the mixture models and the *ρ *established in the previous publication [8] to determine the T_m _categories and mixing proportions of these data (Figure 1). Akaike's information criterion (AIC), a measure of how well a model explains the data, with a penalty for the number of parameters estimated, determined that the T_m _of the four sequences were best represented by a three-category mixture model. This model precisely estimated the mixing proportions of the T_m _into the categories, attributing the correct number of T_m _recordings to each of the four sequences (where two of them shared a category). For an overview of the procedure for using mixture models to analyze T_m _data, see the Methods section.

**Figure 2:**
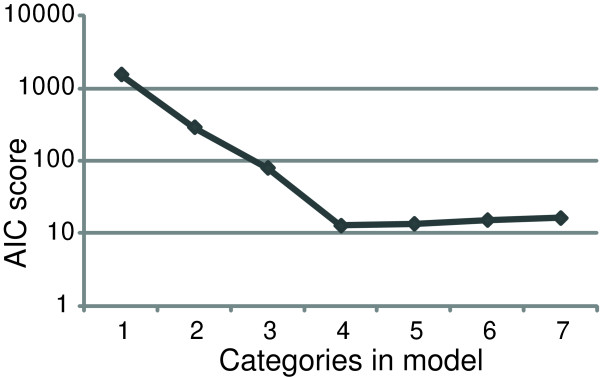
**AIC plotted for models with various numbers of categories.** This AIC plot corresponds to 100 data points generated from four temperatures with 5 × *σ *separations (80, 80.3, 80.6, 80.9). The minimum at four indicates that a model with four categories is optimal. Models with 1–3 categories have a high AIC, indicating that they do not sufficiently explain the data. Models with 5–7 categories do not improve the correlation of the model to the data points sufficiently to justify the additional parameters estimated.

**Figure 3:**
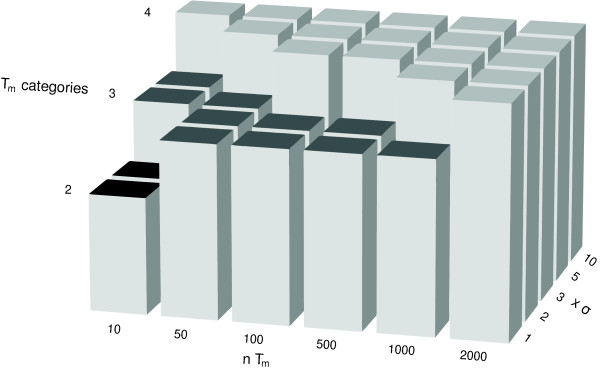
**Plot of the optimal model, as defined by AIC, for simulated data consisting of four T_m _with varying separations against the number of T_m _used to construct the model.** Y-axis labeled "T_m _categories" indicates the number of categories in the model determined to be optimal by AIC. X-axis indicates the number of simulated T_m _data points used in the construction of the model. Z-axis indicates the separation of the T_m _(multiples of *σ*) used to generate the data points.

We next assessed the performance of the mixture model analysis in constructing models for categories of T_m _with varying separations. Therefore, we generated simulated data points mimicking the T_m _of four sequences separated by multiples of *σ*. These data were used to identify the model that best explained the data according to AIC (see an example of the AIC plot in Figure 2) for a range of T_m _separations and numbers of data points (Figure 3). A large separation of T_m_, 10 × *σ *(0.6°C), allow the mixture model analysis to close in on four separate categories with only 10 data points. Smaller separations of T_m _require larger numbers of data points to determine the correct number of T_m _categories. The distinction of categories with a separation of 1 × *σ *required approximately 2000 data points to model the correct number of T_m _categories.

**Figure 4:**
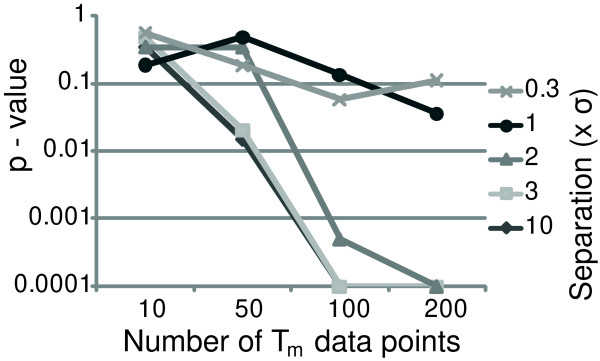
**Plot of *P *values determined with *χ*^2 ^tests against the number of data points fitted to the mixture model.***χ*^2 ^tests between four equally proportioned T_m _categories were compared with the fitted mixing proportions determined from data points when one of the four categories was not represented. The different lines represent the various separations of "temperatures" used to generate the data points, where each line is denoted by a multiplier of *σ*.

Next, we evaluated the fit of the data points to preestablished models. For this purpose, we generated data points corresponding to a sample containing three of four possible T_m _represented in a model. We compared the mixing proportions reported by the mixture model analysis with the mixing proportions in which all four T_m _were present at equal frequencies. In Figure 4, the *P *values obtained from *χ*^2 ^analyses for various separations of the T_m _are plotted against the numbers of data points used. The *P *values for the *χ*^2 ^test drop rapidly with increasing sample numbers for any T_m _separation of more than 1 × *σ*. With smaller separations of the T_m _categories, the mixture model analysis is unable to reliably establish the differences in the mixing proportions.

## Discussion

We report the application of mixture models to the analysis of high-resolution T_m _data. Whereas the plasmid T_m _data reported are sufficiently separated to be stratified manually, we use these data to demonstrate the principle that can be applied to analyze more complex T_m _data.

Mixture model analysis of T_m _data entails the construction of a model based on the T_m _data for a set of primers. With such a model established, it is possible to fit smaller subsets of data to calculate the mixing proportions of the T_m _categories of the model. This gives a proxy marker for the frequency distributions of different amplicon sequences in the analyzed data. This approach requires no prior knowledge of how many different amplicons are present and there is no limit to the number of different T_m _that can be distinguished. However, the T_m _analysis method with mixture models only reports the minimum number of different sequences required to explain the T_m _data because different sequences can have the same T_m_.

Mixture model analysis is a modern type of cluster analysis. The purpose of cluster analysis is to group data that have properties in common. When constructing the mixture model for a set of primers, the number of categories in the model that most appropriately explains the T_m _data is determined by AIC. Other information criteria exist, such as the Bayesian information criterion, but this penalizes free parameters more harshly than does the AIC.

By empirical testing with simulated data, we found that smaller separations of T_m _require exponentially larger numbers of data points to distinguish the correct number of categories in a mixture model. Insufficient numbers of observations yield an underestimation of the numbers of unique T_m _represented by the data, erring on the side of safety. In other words, with insufficient data, the number of unique sequences in the data is underestimated by the optimal model.

In an established model, based on a large number of T_m _observations, a smaller number of observations can be fitted to calculate the mixing proportions in the T_m _categories. These proportions can then be compared between sets of T_m _data as frequency distributions of sequences and analyzed with *χ*^2 ^tests. We observed that, whereas a large number of T_m _observations are required to establish a model with a small separation between categories (e.g., 1000 data points are required with 2 × *σ *separation), far fewer are sufficient for comparisons once the model is established (e.g., 100 data points for *P *< 0.001). A separation of the T_m _categories in the model of less than 1 × *σ *results in unreliable mixing proportions. However, this should rarely be a problem in practice, because constructing the models puts a larger constraint on T_m _separation by AIC. In other words, models constructed with mixture model analysis will consist of T_m _categories separated by more than 1 × *σ*.

Not all dissociation curves are easily defined by a single T_m_, as in the case of multiple domain transitions in longer sequences [9] (generally longer than those generated in real-time PCR assays) and for heterodimers. Using the GcTm approach to curve fitting and SYBR Green I chemistry, such melting profiles will be assigned a single T_m _value. Although some additional information is therefore lost, mixture model analysis still validly identifies clusters of T_m _and sequences. There is an established high-resolution amplicon melting analysis (usually denoted HRM) using LCGreen, primarily based on differences in the profiles of melting curves rather than on absolute T_m _[10]. Although this method is superior to mixture model analysis in identifying heterodimers, absolute T_m _values are required to identify homodimers. Recently, a method with sufficient resolution to distinguish base-pair neutral homozygotes was reported [11]. Mixture model analysis of T_m _can be used in all cases where the T_m _can be denoted as a single value, but primarily for homodimer discrimination.

## Conclusion

In conclusion, the mixture model analysis of T_m _presented here allows the unbiased analysis of high-resolution T_m _data. This analysis is applicable to the identification of sequences in T_m _data regardless of the method by which the T_m _are acquired, provided the measurement error is known. Mixture models allow T_m _analyses to be performed on more complex and varied sequence targets than hitherto possible. Possible applications include typing microbial strains and their relative abundances in a population and the analysis of transcripts containing repetitive elements [3,4,6,12].

## Methods

### Finite mixture models

Mixture models are useful for describing complex populations with observed or unobserved heterogeneity. The term *mixture model *encompasses many types of statistical structures. Here, we use it to denote *mixture distributions*. A mixture distribution is a collection of statistical distributions that arise when mixed populations are sampled that have a different probability density function for each component.

Let *X *be a random variable or vector taking values in sample space *χ *with the probability density function

*g*(*x*) = *π*_1 _*f*_1 _(*x*) + ... + *π*_*k *_*f*_*k *_(*x*),   *x *∈ *χ*,

where 0 ≤ *π*_*i *_≤ 1,   *i *= 1, ..., *k*,   *π*_1 _+ ... + *π*_*k *_= 1.

Such a model can arise if one is sampling from a heterogeneous population that can be decomposed into *k *distinct homogeneous subpopulations, called *component populations*. If these components have been "*mixed*" together, and we measure only the variable *X *without determining the particular components, then this model holds. We say that *X *has a finite mixture distribution and that *g*(·) is a finite mixture density function. The parameters *π*_1_,..., *π*_*k *_are called *mixing weights *or *mixing proportions*, and each *π*_*i *_represents the proportion of the total population in the *i-*th component.

There is no requirement that the component densities should all belong to the same parametric family, but in this paper, we keep to the simplest case where *f*_1_(*x*),..., *f*_*k*_(*x*) have a common functional form but different parameters.

We apply the theory of finite mixture models to T_m _data consisting of normally distributed components in a mixture model, where each component has a standard deviation of *σ*°C. The finite mixture density function is then as follows:

$$g(x|\psi )=\sum_{i=1}^{k}\pi_{i}\frac{1}{\sigma \sqrt{2\pi }}\exp\left\{\frac{{\left(x-\mu_{i}\right)}^{2}}{2\sigma^{2}}\right\}$$

where **ψ **= (*π*_1_,..., *π*_*k*_, *μ*_1_,..., *μ*_*k*_, *σ*)^*T*^.

The likelihood function corresponding to the data (*x*_1_,..., *x*_*n*_) is as follows:

$$L(\psi ;x_{1},\ldots x_{n})=\prod_{j=1}^{n}g(x_{j}|\psi ).$$

The logarithm of the likelihood function is

$$\ln L(\psi )=\sum_{j=1}^{n}\ln g(x_{j}|\psi ).$$

We attempt to find the particular **ψ **that maximizes the likelihood function. This maximization can be undertaken in the traditional way by differentiating *L*(**ψ**; ×) with respect to the components of **ψ **and equating the derivatives to zero to give the likelihood equation:

$$\frac{\partial L\left(\psi \right)}{\partial \psi }=0,\text{ or equivalently}\frac{\partial \ln L(\psi )}{\partial \psi }=0.$$

Quite often, the log likelihood function cannot be maximized analytically, i.e., the likelihood equation has no explicit solutions. In such cases, it is possible to compute the maximum likelihood of **ψ **iteratively. To calculate maximum likelihood estimates, we use the expectation maximization (EM) method in combination with the Newton-Raphson algorithm. Iterations of the EM algorithm consist of two steps: the *expectation step *or the *E-step *and the *maximization step *or the *M-step *[13,14]. The Newton-Raphson algorithm for solving the likelihood equation approximates the gradient vector of the log likelihood function by a linear Taylor series expansion [15]. We use the Newton-Raphson algorithm in the M-step of the EM method.

We developed an algorithm that allows the automated estimation, in parallel, of a finite number of normally distributed components. The number of components can be assessed by several different methods, although none of them is optimal. We chose the AIC [16,17]. AIC is a relative score between different models where the selection of the optimal model is made by considering the number of data points and categories and the separation of the T_m _categories. AIC is defined as -2*L*_*m *_+ 2*m*, where *L*_*m *_is the maximized log likelihood and *m *is the number of parameters.

### Acquisition of HERV-W gag T_m_

T_m _data were generated with GcTm, as previously described [8], on dissociation data obtained from the amplification of plasmids containing known HERV-W *gag *sequences.

Simulated T_m _data recordings and GcTm analysis were performed in MATLAB™ (The MathWorks) version 7.0.1.24704 with the Optimization Toolbox. Mixture model analysis was performed in R 2.6.0 [18] with the MIX software [19,20].

### Overview of mixture model analysis of T_m_

A mixture model is constructed for a set of primers. The model should be constructed on a large enough sample of T_m _data to expect all possible sequences to be represented. The T_m _data are then stratified into small-interval groups and the frequency distributions of these arbitrary categories are used to construct and compare the mixture models. AIC is used to evaluate which model best explains the data, while a minimum number of different categories is used. Lower values of AIC indicate the preferred model, i.e., the one with the fewest parameters. Once a model is selected, T_m _data from different samples can be fitted to the model and the mixing proportions compared between samples. Differences between samples can be evaluated with *χ*^2 ^tests if a conservative stance is taken, depending on the separation between the T_m _categories and the numbers of data points.

## Abbreviations

T_m_: Melting temperature; AIC: Akaike's information criterion; HERV: human endogenous retrovirus; EM: expectation maximization.

## Authors' contributions

CN conceived the study, tested and prepared the manuscript; FU developed the method and critically revised the manuscript; JT developed the method and prepared the manuscript; HK conceived the study and prepared the manuscript.
